# Case Report: Recurrent sinus arrest induced by repeated vomiting

**DOI:** 10.3389/fphys.2026.1732019

**Published:** 2026-03-02

**Authors:** Zhongbiao Jiang, Yudong Zhang, Zhihong Wu, Xuan Jiang, Lin Hu, Shuang Zhang, Mingxian Chen

**Affiliations:** 1 Department of Radiology, The Second Xiangya Hospital of Central South University, Changsha, Hunan, China; 2 Medical Simulation Center, Guilin Hospital of the Second Xiangya Hospital, CentralSouth University, Guilin, Guangxi, China; 3 Department of Cardiology, The Second Xiangya Hospital of Central South University, Changsha, Hunan, China

**Keywords:** autonomic dysfunction, neuromyelitis optica spectrum disorder, pacemaker, scope, sinus arrest

## Abstract

Neuromyelitis optica spectrum disorder (NMOSD) can affect autonomic centers in the dorsal medulla, but presentation with recurrent sinus arrest is extremely rare. We report a 71-year-old woman with 10 days of persistent hiccups and vomiting who developed repeated syncopal episodes. ECG monitoring revealed vomiting-induced bradycardia progressing to sinus arrest, and Holter monitoring showed nocturnal sinus pauses up to 40.6 s. Routine cardiac and neurological evaluations were unremarkable, but serum anti–aquaporin-4 antibodies were markedly elevated, and brain MRI demonstrated a dorsal medullary lesion, confirming NMOSD with area postrema involvement. The patient was treated with intravenous immunoglobulin and high-dose methylprednisolone, resulting in complete resolution of vomiting and normalization of sinus node function. A temporary pacemaker was required acutely, and a permanent pacemaker was implanted before discharge. She remained stable on rituximab without relapse. This case underscores that severe vomiting-triggered sinus arrest may be an autonomic manifestation of NMOSD and is reversible with timely immunotherapy.

## Introduction

Neuromyelitis optica spectrum disorder (NMOSD) is an autoimmune astrocytopathy characterized by severe, relapsing inflammatory attacks primarily affecting the optic nerves and spinal cord (Uzawa et al., 2024). With the identification of anti–aquaporin-4 (AQP4) antibodies and the expansion of diagnostic criteria, it is now recognized that lesions may also involve the brainstem, particularly the area postrema of the dorsal medulla. Area postrema syndrome—manifesting as intractable nausea, vomiting, and hiccups—is a well-established nonclassical presentation of NMOSD (Su and Wang, 2024).

Beyond gastrointestinal symptoms, involvement of the dorsal medulla may impair autonomic cardiovascular regulation. Although autonomic dysfunction in NMOSD is increasingly appreciated, clinically significant bradyarrhythmias and sinus node dysfunction remain exceedingly rare. Only a few cases have described sick sinus syndrome or sinus arrest attributed to NMOSD, often mimicking primary cardiac disease and posing substantial diagnostic challenges (Lin et al., 2023; Hamaguchi et al., 2022).

Here, we describe an unusual case in which recurrent vomiting triggered profound sinus arrest leading to repeated syncope and documented sinus arrest. The patient was ultimately diagnosed with NMOSD with dorsal medullary involvement, and her cardiac abnormalities resolved following timely immunotherapy. This case highlights the importance of considering NMOSD in patients presenting with otherwise unexplained vomiting-associated syncope or severe bradyarrhythmias, and it underscores the potential reversibility of autonomic cardiac dysfunction with appropriate treatment.

## Interpretation

**FIGURE 1:**
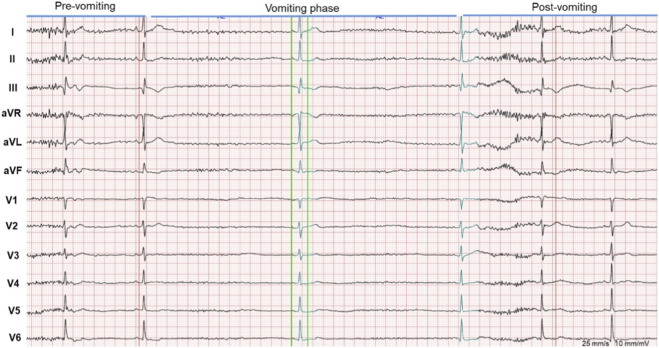
Monitoring ECG. It presents vomiting-related bradycardia and sinus arrest. ECG monitoring shows a significant heart rate decrease during vomiting, progressing to sinus arrest with syncopal episodes, followed by gradual heart rate recovery.

**FIGURE 2:**
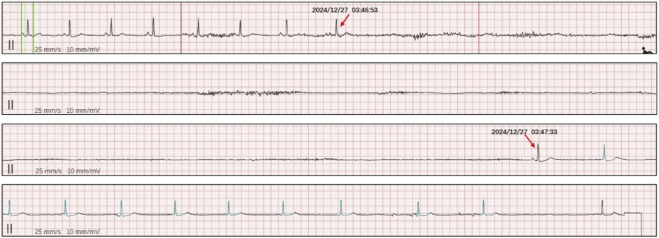
Holter ECG. It shows sinus arrest with the longest RR interval at about 40.6 s during sleep.

In Figure 1, the ECG monitoring presented vomiting-related bradycardia and sinus arrest. ECG monitoring shows a significant heart rate decrease during vomiting, progressing to sinus arrest with syncopal episodes, followed by gradual heart rate recovery. Holter monitoring in Figure 2 showed sinus arrest with the longest RR interval at about 40.6 s during sleep.

## Case presentation

A 71-year-old woman presented with a 10-day history of persistent hiccups and repeated episodes of vomiting. She had initially been evaluated at a local hospital, where she was diagnosed with reflux esophagitis, but her symptoms progressed. Six hours before admission to our hospital, she experienced five episodes of witnessed syncope, each accompanied by brief limb jerking and upward eye deviation lasting 5–10 s. All episodes occurred while she was seated. She was admitted to the neurology department with a preliminary suspicion of epilepsy.

During hospitalization, continuous electrocardiographic monitoring revealed marked bradycardia occurring immediately after vomiting (Figure 1), followed by sinus arrest and transient loss of consciousness, with spontaneous recovery of heart rate thereafter. Additional monitoring captured sinus pauses lasting approximately 9 s. Twenty-four-hour Holter monitoring demonstrated recurrent episodes of sinus arrest during sleep, with the longest RR interval measuring 40.6 s (Figure 2).

**FIGURE 3:**
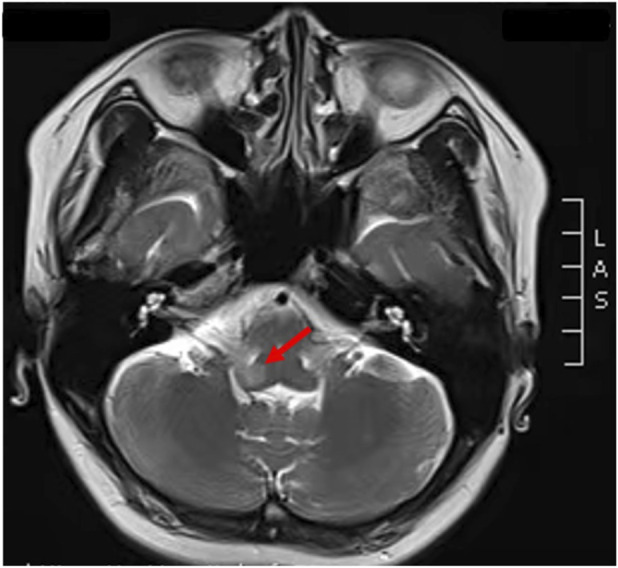
Brain MRI of the patient with neuromyelitis optica spectrum disorder. Brain MRI T2-weighted images demonstrate hyperintense lesions in the dorsal medulla oblongata (red arrows).

The patient’s prior medical history was unremarkable, and she was not taking medications known to induce bradyarrhythmias. Initial laboratory tests, electrolytes, cardiac enzymes, echocardiography, electroencephalography, and abdominal CT scans were normal. However, immunological testing showed elevated anti–aquaporin-4 antibodies and strongly positive anti–SS-A antibodies (+++). Cerebrospinal fluid analysis was normal. Brain MRI revealed a hyperintense lesion in the dorsal medulla oblongata, consistent with involvement of the area postrema (Figure 3).

Therefore, this patient fulfils the current diagnostic criteria for NMOSD by presenting with a core clinical characteristic (area postrema syndrome), corresponding brainstem MRI lesions, and supportive immunological findings, confirming a definitive diagnosis of NMOSD rather than an “NMOSD-like” syndrome. The patient was diagnosed with symptomatic sick sinus syndrome secondary to NMOSD. A temporary pacemaker was implanted due to recurrent sinus arrest. She subsequently received intravenous immunoglobulin (0.4 g/kg/day for 5 days) and high-dose methylprednisolone (1000 mg/day for 5 days). Her vomiting and hiccups resolved rapidly, and sinus node function normalized. After removal of the temporary pacemaker, her heart rate and blood pressure stabilized at 66 bpm and 110/70 mmHg, respectively. Before discharge, a permanent pacemaker was implanted for long-term rhythm support. She was transitioned to rituximab for maintenance immunotherapy and remained clinically stable without recurrence of neurological or cardiac symptoms. During 12-month follow-up period, the patient remained clinically stable without neurological relapse, recurrent vomiting, syncope, or sinus arrest.

## Discussion

This case illustrates an uncommon but clinically significant manifestation of NMOSD: recurrent vomiting-induced sinus arrest leading to repeated syncope and documented sinus arrest (Pittock et al., 2021). Although gastrointestinal symptoms such as intractable nausea, vomiting, and hiccups are characteristic of area postrema syndrome (APS), profound bradyarrhythmias and sick sinus syndrome (SSS) represent rare extensions of dorsal medullary involvement. Only a handful of reports have described NMOSD presenting with sinus node dysfunction, and most cases were initially misinterpreted as primary cardiac disease (Teoh et al., 2024). Our case expands the existing literature by demonstrating that vomiting itself, triggered by APS, can precipitate extreme vagal activation and unmask central autonomic instability, ultimately resulting in prolonged sinus arrest.

The dorsal medulla, particularly the area postrema and nucleus tractus solitarius (NTS), plays a central role in integrating autonomic cardiovascular control. Lesions in this region, as commonly observed in AQP4-positive NMOSD, can disrupt baroreflex pathways, impair sympathetic output, and lead to excessive parasympathetic activation. Previous case reports have shown that medullary lesions may produce bradycardia, sinus pauses, atrioventricular block, or even asystole. The two published cases of NMOSD-associated SSS similarly documented significant sinus pauses and highlighted the potential for reversible autonomic dysfunction following immunotherapy (Lin et al., 2023). Consistent with these findings, our patient exhibited vomiting-induced bradycardia progressing to sinus arrest and nocturnal episodes of sinus arrest exceeding 40 seconds—features that cannot be explained by intrinsic sinus node disease alone.

A unique aspect of this case is the clear temporal association between vomiting and sinus arrest. Vomiting can activate vagal afferents and transiently suppress sinus node automaticity, but such responses are rarely severe or sustained (Kwon et al., 2020; Miller and Leslie, 1994). In our patient, the extreme degree of vagal hyperactivity likely reflected the combination of APS-induced persistent vomiting and disrupted autonomic regulation from the dorsal medullary lesion. The rapid reversal of both gastrointestinal symptoms and sinus node dysfunction after immunotherapy strongly supports an NMOSD-related mechanism rather than primary degenerative SSS. This parallels observations from other reports in which bradyarrhythmias improved after high-dose steroids, plasmapheresis, or IVIG (Cree et al., 2024; Levy et al., 2021).

Temporary pacing is essential in NMOSD patients with severe bradyarrhythmias to prevent sudden cardiac death during the acute inflammatory phase. However, the decision to implant a permanent pacemaker remains controversial (Lin et al., 2023; Teoh et al., 2024). Some reported cases demonstrated full recovery of sinus node function after immunotherapy, while others experienced persistent dysfunction or recurrent pauses. Considering our patient’s documented 40.6-s sinus arrest and multiple syncope-associated asystolic events, permanent pacing was deemed appropriate despite clinical improvement. Given the potential for relapse in NMOSD and the unpredictable course of medullary involvement, long-term rhythm protection may offer an additional safety margin.

This case underscores several key lessons. First, clinicians should maintain high suspicion for NMOSD in patients presenting with otherwise unexplained vomiting, hiccups, or visceral autonomic symptoms, particularly when accompanied by bradyarrhythmias or syncope (Huda et al., 2019). Second, medullary lesions can produce life-threatening cardiac arrhythmias through central mechanisms rather than primary cardiac pathology. Third, timely initiation of immunotherapy is crucial, as autonomic dysfunction is potentially reversible with appropriate treatment. Finally, multidisciplinary collaboration between neurology and cardiology is essential for early diagnosis, prevention of sinus arrest, and long-term disease management.

In conclusion, vomiting-induced sinus arrest may be an underrecognized manifestation of NMOSD with area postrema involvement. Prompt recognition and immunotherapy can reverse autonomic cardiac dysfunction and prevent fatal outcomes. This case reinforces the importance of considering NMOSD in the differential diagnosis of episodic bradyarrhythmias associated with medullary symptoms.

## Data Availability

The original contributions presented in the study are included in the article/supplementary material, further inquiries can be directed to the corresponding author.
